# Supplementary material for: Domain adaptive learning for multi realm sentiment classification on big data
Source: PLoS One. 2024 Apr 1;19(4):e0297028. doi: 10.1371/journal.pone.0297028 (PMC10984522; doi:10.1371/journal.pone.0297028)
Supplement: Attachment — Submitted filename: Response Sheet.docx [file pone.0297028.s001.docx]

**Manuscript ID:  PAPER ID: PONE-D-23-20322**

**Reviewer Comments, Author Responses and Manuscript Changes**

We are very grateful for the reviews provided by the editors and each of the external reviewers of this manuscript titled “**Domain Adaptive Learning for Multi Realm Sentiment Classification on Big Data”**. The comments are encouraging, and we thank you for giving us the opportunity to resubmit our research work. The suggestions offered by the reviewers have been immensely helpful, and we have responded to them individually, indicating precisely how we addressed each concern or problem and describing the changes we have made. Please see below for our detailed response to the comments. All section numbers refer to the updated manuscript file with tracked changes.

**Responses to Editorial Comments (Journal Requirements)**

**Comment 1:**

Please note that funding information should not appear in the Acknowledgments section/Funding section or Any other areas of your Manuscript. We will only publish funding information present in the Funding Statement section of the online submission form. Please remove any funding-related text from the manuscript.

**Response:**

Thank you very much. We have revised the acknowledgment section as per your guidance. The funding-related text has been removed from the manuscript, and the study's support from King Saud University is mentioned in the acknowledgment.

We have observed a recently published paper in PLOS ONE with a similar acknowledgment statement, which can be found at the following link for your reference

https://journals.plos.org/plosone/article?id=10.1371/journal.pone.0289410#ack

**Responses to Reviewer 1**

**Comment 1:**

***The paper highlighted the benefits of transfer learning concept for Multi domain Sentiment classification (MDSC) technique on BIG DATA. Transform learning Models like BERT, ULMFiT etc. have been used to study the analysis. Various reliable Sentiment Analysis Datasets were used to influence the outputs.A thorough Literature Survey of the various transfer learning methods were also done which added a feather to the work done .Analysis using metrics like accuracy & F1 score was done.***

The work can be extended with more relevant metrices and inculsion of statistical analysis will take the wok to a high level.

**Response:** Thank you for the approval. According to the reviewer’s comment, the answer is as follows:

Thank you for pointing this out. In general, if you are working with an imbalanced dataset where all classes are equally important, using the macro average would be a good choice as it treats all classes equally. If you have an imbalanced dataset but want to assign greater contribution to classes with more examples in the dataset, then the weighted average is preferred. This is because, in weighted averaging, the contribution of each class to the F1 average is weighted by its size. Suppose we have a balanced dataset and want an easily understandable metric for overall performance regardless of the class. In that case, we can go with accuracy, which is essentially our micro F1 score.

**Responses to Reviewer 2**

**Comment 1:**

***The authors used various Bert based semantic encoding mechanisms, however to make the methods clearer to the readers, it will be better to provide a complete graphical framework of the these methods to illustrate their mechanisms.***

**Response:** Thank you for bringing this to our attention.

We employed five distinct datasets as well as transfer learning models such as BERT, RoBERTa, ELECTRA, and ULMFiT. We use that Models Graphical Framework to demonstrate its mechanism.

**Comment 2: *I did not found any cross validation test for model training.***

**Response:**

Following are the reasons for not using cross-validation test with transformers (or deep learning).

**Transfer Learning Effectiveness:** Transfer learning, especially in the context of pre-trained transformer models like BERT, GPT-3, and their variants, has been remarkably effective in a wide range of natural language understanding tasks. These models are typically pre-trained on massive datasets containing a diverse range of text, making them strong feature extractors. Fine-tuning these pre-trained models on task-specific data often yields excellent results, and this approach reduces the need for extensive cross-validation. With transfer learning, you can effectively leverage pre-trained weights and fine-tune them on your specific task with a relatively small dataset.

**Large Datasets:** When you have a substantial amount of data, the impact of selecting a bad train/test split becomes less significant. With a small dataset, a poor split could lead to overfitting or under-fitting, making cross-validation crucial for assessing model performance. However, with large datasets, you can often afford to set aside a substantial portion for training and still have a reasonable-sized test set for evaluation.

**Comment 3: The author should provide complete Hyper-parameters for model training in a tabular form, authors may increase epochs for better training results.**

**Response: SECTION: 3.3.4 HYPERPARAMETERS**

The Hyper-parameters for each model training with fields Learning Rate, Batch-Size, Epochs and MSL has been mentioned in table 7.

The hyper parameters can be adjusted to produce the best outcomes. These variables need to be manually adjusted because the model itself cannot learn them. The term "hyperparameters" refers to a wide range of variables, such as the learning rate, training batch size, dropout rate, number of training epochs, and any other optimizer-related variables that can be altered, like the Adam optimisation technique. We employ bert-base-cased for BERT [46], roberta-base for RoBERTa [64], and google/electra-base-discriminator for ELECTRA [65] as baselines for deep neural network techniques.

**Table 7: HYPERPARAMETERS EMPLOYED FOR BERT, ROBERTA, AND ELECTRA**

**Responses to Reviewer 3**

**Comment 1: The statistical indicators macro-average F1 score and micro-average F1 score has mentioned in equation 3 and 4 but results were reported of F1 score only. Need to be clarify.**

**Response:**

We mentioned weighted average F1 score. If you have an imbalanced dataset but want to assign greater contribution to classes with more examples in the dataset, then the weighted average is preferred. This is because, in weighted averaging, the contribution of each class to the F1 average is weighted by its size. Suppose you have a balanced dataset and want an easily understandable metric for overall performance regardless of the class. In that case, you can go with accuracy, which is essentially our micro F1 score.

**Comment 2: Write down limitations in current literature.**

**Response:**

We agree with the reviewer’s assessment. Accordingly, throughout the manuscript, we have revised the literature review according to the current literature and added limitations of the work.

**Comment 3: Which split ratio you used for training/testing division?**

**Response:**

We’ve used five (5) different datasets and in three of them we used four (4) different samples size for training. And for each sample we have used 70:30 split ratio.

**Comment 4: Several typos and grammatical errors are found.**

**Response:** The grammatical errors have been corrected on [insert the exact location where the change can be found in the revised manuscript].

**Comment 5: Some paragraphs are too long to read. The authors should try for readability and comprehensibility by dividing paragraphs into two or more.**

**Response:**

The partition of paragraphs has been corrected accordingly.

**Comment 6: The conclusion section is weak. The conclusion section needs significant revisions. It should briefly describe the findings of the study and some directions for further research**.

**Response:**

This research demonstrated contemporary transfer learning models in multi-realm sentiment classification. This research focuses on evaluating the impact of transfer learning on sentiment analysis results and investigating the effectiveness of domain adaptation. The study employs well-known transfer learning models such as BERT, RoBERTa, ELECTRA, and ULMFiT to enhance the performance of sentiment analysis. Various transformer models are used to analyze sentiment, and the performance of LSTM and CNN is compared.

Based on relevant research, we went with techniques that do not necessitate pretraining the whole model from scratch, despite resetting the model’s weights using an existing one. It is a practical method for a plethora of reasons: First, when the quantity of accessible textual data is insufficient to meet the requirements for pretraining a completely new model. Second, the computational time, although still significant and depends on the quantity of extra training data, is substantially less than typical pretraining, which would take a week. The primary goal of this study is to address the challenge of limited labeled data for predicting sentiment polarity in a target domain by using a classifier trained on a source domain. This research explores the use of fine-tuned transformers and the highly modular Framework for Adapting Representation Models (FARM) to model sentiment analysis from text.

The pretrained models BERT, RoBERTa, and ELECTRA are employed, which are subsequently fine-tuned with FARM to represent the downstream assessment to identify sentiments, along with ULMFiT. Experiments are conducted on five publicly available sentiment analysis datasets, namely Hotel Reviews (HR), Movie Reviews (MR), Sentiment140 Tweets (ST), Citation Sentiment Corpus (CSC), and Bioinformatics Citation Corpus (BCC). LSTM performs better than CNN in most domains when the results are compared to deep learning models like CNN. FARM framework-based models extensively beat deep learning techniques. Other domain adaption situations, such as named entity recognition, question answering, and reading comprehension, might benefit from the proposed post-training technique. In future work, we intend to further assess our approach for these domain adaptation challenges and to explore other pretrained models.

**Comment 7: Following research papers can be considered in your manuscript for possible references:**

**Response:**

It would have been interesting to explore this aspect. we agree that this is an important consideration in this manuscript.
